# Maternal pre-pregnancy weight status modifies the influence of PUFAs and inflammatory biomarkers in breastmilk on infant growth

**DOI:** 10.1371/journal.pone.0217085

**Published:** 2019-05-29

**Authors:** Henry Nuss, Abby Altazan, Jovanny Zabaleta, Melinda Sothern, Leanne Redman

**Affiliations:** 1 Behavioral and Community Health Sciences, School of Public Health, Louisiana State University Health, New Orleans, Louisiana; 2 Pennington Biomedical Research Center, Baton Rouge, Louisiana; 3 Department of Pediatrics, School of Medicine, Louisiana State University Health, New Orleans, Louisiana; State University of New York at Buffalo, UNITED STATES

## Abstract

**Background:**

Human breastmilk contains pro- and anti-inflammatory compounds and hormones that can influence infant growth. However, little is known about the specific interrelationships between these compounds and whether their effects on infant growth may be influenced by pre-pregnancy weight status.

**Objective:**

The purpose of this novel, prospective cohort study was to assess the interrelationships between pro-inflammatory cytokines (TNF-α, IL-6), hormones (insulin, leptin) and PUFAs (n-6, n-3) in blood and breastmilk in early postpartum between women with normal BMI (G**roup 1**, n = 18; 18.5<BMI≤24.9 kg/m^2^) and with overweight/obesity (G**roup 2**, n = 15; BMI≥25.0 kg/m^2^) before pregnancy to determine if these components correlated to infant growth measures at age 4–8 weeks.

**Methods:**

Participants were robustly phenotyped along with their infants at 4–8 weeks postpartum. TNF-α, IL-6, insulin, leptin, and n-3 and n-6 PUFAs measured in blood and breastmilk and compared between pre-pregnancy BMI groups and with infant weight, length, head circumference and % fat mass.

**Results:**

Group 1 women had higher serum leptin (p<0.01) and breastmilk leptin (p<0.001) compared to Group 2. Other inflammatory markers, hormones, and total n-6, n-3 and n-6/n-3 ratio PUFAs were similar between pre-pregnancy BMI groups. No relationships were observed between whey inflammatory markers, hormones, PUFAs and growth measures in infants born to Group 2 women. However, TNF-α was positively related and, IL-6, leptin, insulin, total n-6, n-3 and n-6/n-3 PUFAs in whey breastmilk were negatively correlated to infant growth measures in infants born to Group 1 women (p<0.01).

**Conclusions:**

Pro-inflammatory qualities of breastmilk were associated with infant growth measures regardless of maternal pre-pregnancy BMI. However, infants born to women with overweight or obesity demonstrated less responsive growth to breastmilk contents. More studies are needed to assess longitudinal effects of this impact.

## Introduction

Childhood obesity rates in the U.S. have increased significantly in recent decades [1–3]. Many studies have shown that breastfeeding may be protective against excessive weight gain during early life [4–9]. However, the precise relationship between breastfeeding and early weight gain is not well understood [5]. Recently studies have implicated specific bioactive compounds in human breastmilk which may have varying degrees of impact on infant growth. Hormone such as insulin and leptin, known for their roles in blood glucose and energy homeostasis (respectively), have been well documented in breastmilk, yet their influence on infant growth is not well established. Pro-inflammatory cytokines, such as tumor necrosis factor alpha (TNF-α) and interleukin six (IL-6), have often been associated with obesity [5, 10, 11]. Both cytokines are found in breastmilk, yet there are few studies which have assessed whether they influence infant growth and the results are inconsistent [9, 12–14].

Polyunsaturated fatty acids present in breastmilk may also exert an influence on the relative concentrations of TNF-α and IL-6. Some long-chain omega-3 (n-3) polyunsaturated acids (PUFAs) are often associated with anti-inflammatory properties [15, 16]. A growing body of evidence suggests that n-3 PUFAs such as eicosapentaenoic acid (EPA) and docosahexaenoic acid (DHA) may reduce circulating TNF-α and IL-6 [17–19]. Conversely, higher levels of omega-6 (n-6) PUFAs such as arachidonic acid (AA) are shown to have pro-inflammatory properties [19] and associate with increased levels of IL-6 and TNF-α in pregnant women [20]. While there may be some association between n-6 PUFAs in maternal blood and infant body composition [21], no studies have explored whether the mechanism by which maternal PUFAs influence infant body composition is through an improved inflammatory (lower IL-6 and TNF-α) profile in breastmilk. Based on the relationship between PUFAs and inflammatory markers in blood, it is plausible that the relative proportions of n-3 and n-6 PUFAs in breastmilk also influence TNF-α and IL-6 in breastmilk and with effects on infant growth.

Much of aforementioned information that has been published on this emerging field of lactational programming has come from multiple studies of independent cohorts which have assessed one or more of the milk components previously mentioned. None, however, have assessed the interrelationships between pro-inflammatory cytokines, hormones and PUFAs on infant growth in the same sample at the same time. Therefore, the objective of this novel, prospective cohort study was to observe and describe any interrelationships between milk concentrations of inflammatory markers (TNF-α, IL-6), hormones (insulin, leptin), and n-3 and n-6 PUFAs on infant growth measures at four to eight weeks postpartum. We also conducted comparison analyses according to mother’s pre-pregnancy body mass index (BMI) between breastfeeding women without overweight/obesity (BMI < 25, herein referred to as **Group 1**) those with overweight/obesity (BMI ≥ 25, herein referred to as **Group 2**).

## Materials and methods

### Study design

The Mom2Baby Pilot study (NCT02317653) was a prospective cohort study in 33 pregnant women at Pennington Biomedical Research Center. Participants completed a study visit in early postpartum (4–8 weeks). The study visit was performed in the morning following an overnight fast, and included anthropometric measurements and collection of a fasting blood sample and a breastmilk sample for measurement of inflammatory markers and hormones (insulin, leptin, TNF-α, and IL-6) and PUFAs. Infants born to mothers in the study received weight, length, head circumference, and body composition measurements at 4–8 weeks of age.

### Participants and recruitment

Individuals were recruited through targeted advertisements, emails and through referrals by local obstetricians [22]. Participants were eligible if they were English speaking, 18 to 40 years, pregnant and carrying a viable singleton fetus, with no gestational diabetes diagnosis, and intended to breastfeed or provide breastmilk to their infant for at least the first 2 months of age. Women were classified according to pre-pregnancy BMI as Group 1 (BMI = 18.5–24.9 kg/m^2^) or Group 2 (BMI ≥ 25.0 kg/m^2^). The study was approved by Pennington Biomedical Institutional Review Board and Louisiana State University Health Sciences Center and written informed consent was obtained by all participants prior to the initiation of procedures. Group 2 women were recruited as part of the Expecting Success study (NCT01610752) from February 2013 to March 2014, and Group 1 women were recruited for the Mom2Baby Pilot study from February 2015 to September 2015.

### Maternal demographics and anthropometrics

Sociodemographic data including age, race, marital status, household income, level of education, and living situation were obtained from self-reported questionnaires. Gestational age was calculated using the self-reported last menstrual period date and was confirmed from prenatal medical records. Height was measured twice using a wall-mounted stadiometer with head in the Frankfort position. Pre-pregnancy BMI was calculated using self-reported pre-pregnancy weight and height measured in the study.

### Biospecimen collection and breastfeeding practices

Approximately 30 mL of whole blood was collected from an antecubital vein for blood chemistry analysis. For breastmilk, participants obtained a breastmilk sample from a single extraction of one breast using an electronic breast pump after at least a 2-hour fast. Samples were collected at home on the day of the visit and were stored on ice for transportation to the clinic. Upon arrival to clinic, a portion of the homogenized whole breastmilk was aliquoted, and the remaining homogenized whole breastmilk was centrifuged at 3750 RPM for 15 minutes at 4°C to separate lipid and whey. Lipid and whey samples were aliquoted and frozen at -80°C until analysis. Insulin (EZHIASF-14K, EMD Millipore, Billerica, MA), leptin (DLP00, R&D Systems, Minneapolis, MN), TNF-α (DTA00C, R&D Systems, Minneapolis, MN), and IL-6 (Q6000B, R&D Systems, Minneapolis, MN) were measured in serum and breastmilk whey in duplicate using commercially available immunoassay kits. Lipidomic analyses were completed by the Metabolomics Core at the University of Michigan using standard procedures. Total lipids were extracted from plasma and homogenized whole breastmilk [23], and the methyl ester derivative of total lipids were prepared purified by thin-layer chromatography and analyzed by gas chromatography with the Agilent GC model 6890N with FID detector using HP 88 column. Lipid extraction and gas chromatography provided a total lipid fatty acyl profile including total n-3 PUFAs, total n-6 PUFAs, individual n-3 PUFAs (18:3, 18:4, 20:4, 20:5, 22:5, 22:6) and individual n-6 PUFAs (18:2, 18:3, 20:2, 20:3, 20:4, 20:5, 22:2, 22:4, 22:5). At the time of collection, mothers were asked if they were still currently breastfeeding. Mothers were also if they had ever fed their infants any other substance other than breastmilk and how old (in weeks) their baby was when he/she started to regularly consume other milk (e.g., cow) formula, juice, tea or other fluids or cup mixed with solid foods.

### Infant assessments

Body weight was measured with the infant nude to the nearest 5 grams on a calibrated scale (SCALE-TRONIX, White Plans, NY). Length was measured twice with the infant’s head in the Frankfort position using an infantometer with a stationary headboard and a moveable footboard. Head circumference was measured by a standard measuring tape around the most prominent part of the infant’s head just above the supraorbital ridges. Fat mass and fat free mass were assessed using air displacement plethysmography (PEA POD, COSMED, Concord, CA) with the infant wearing a head cap to cover the hair.

### Statistical considerations

All analyses were completed using SPSS Inc. software, Version 24, released for Windows in 2016 (IBM Corp, Armonk, NY). All tests were performed with significance level α = 0.05, and findings were considered significant when p<α. Participant characteristics were summarized as means and standard deviations for continuous variables or counts and percentages for categorical variables for each group: women without overweight or obesity and women with overweight or obesity. Descriptive statistics were used to describe mean, range and distribution values of all PUFAs and inflammatory markers and hormones in serum, plasma, and breastmilk. Total n-3, total n-6, and individual PUFAs were reported as the percent weight of total fatty acids, respectively. The Shapiro-Wilk test [24] was applied to test for normal distributions among measured values for individual PUFAs and inflammatory markers and hormones (leptin, insulin, TNF-α, and IL-6) in blood and breastmilk. Log transformations were applied to the non-normally distributed variables for leptin, insulin, TNF-α and IL-6 in serum blood and whey breastmilk, as well as several n-3 PUFAs (18:4, 20:4, 20:5, 22:5) and n-6 PUFAs (18:2, 20:2, 20:3, 20:5, 22:2, 22:4, 22:5). Independent sample t tests were used to compare PUFAs and inflammatory markers and hormones by pre-pregnancy BMI and biospecimen type (plasma or serum and breastmilk).

The analysis of covariance (ANCOVA) statistic was used to determine if differences existed between infant growth measures (weight, length, head circumference, and % fat mass) according to weight groups with infant age at time of measurement as a covariate. Linear regressions were performed to calculate associations between PUFAs (total n-3, n-6, n-6/n-3 ratios, and individual PUFAs) and inflammatory markers and hormones (leptin, insulin, TNF-α, and IL-6) in blood and breastmilk. Using infant age in days at 4–8 weeks visit as a covariate, linear regressions were also performed to calculate associations between the studied analytes in breastmilk (total n-3, n-6, n-6/n-3 ratios, individual PUFAs, leptin, insulin, TNF-α, and IL-6). The false discovery rate method known as the Benjamini-Hochberg procedure was applied to minimize type one errors when multiple linear regressions were performed with insulin, leptin, TNF-α, IL-6, individual PUFAs, and infant growth measures [25].

## Results

### Study participants

Thirty-three women enrolled and completed the study. The mean age at enrollment was 29.9 ± 4.1 years, and over 90% of the cohort was Caucasian (Table 1). The majority of women were married or living with a significant other and 90% had a college degree or postgraduate education. More than 75% of households earned at least $50,000 in the past year with an average number of household occupants of 2.9±1.2. There were no differences in demographic information between Group 1 and Group 2 women except for pre-pregnancy BMI, as expected (p<0.001). All of the mothers reported that they were breastfeeding at the time of breastmilk sample collection. Eight mothers responded that they had ever fed their infant something other than breastmilk, such as other milk, formula, juice, tea or cup mixed with solid foods. Seven of those were in Group 2 (BMI ≥ 25), four of which said supplemental feeding was not a regular practice.

**Table 1:** Characteristics of adult, female study participants by pre-pregnancy body mass index (BMI) groups (N = 33).

	BMI < 25	BMI ≥ 25	Statistic
**No**.	18	15	
**Age (years)**	29.0 ± 3.1	30.9 ± 5.0	t = -1.3, p = 0.20
**Pre-pregnancy BMI, kg/m2**	22.7 ± 1.8	29.8 ± 4.0	t = -6.9, p < 0.001
**Pre-pregnancy BMI Groups, n (%)**			
BMI<25	18 (100.0)	0 (0)	χ2 = 33.0
25≤BMI<30	0 (0)	8 (53.3)	df = 2
BMI≥30	0 (0)	7 (46.7)	p < 0.001
**Race, n (%)**			
Caucasian	16 (88.9)	14 (93.3)	χ^2^ = 0.27
African American	0 (0.0)	0 (0.0)	df = 1
Other/More than one race	2 (11.1)	1 (6.7)	p = 0.60
**Education, n (%)**			
1–3 Years College	1 (5.6)	2 (13.3)	χ2 = 3.67
College Degree	5 (27.8)	8 (53.3)	df = 2
Post-graduate Education	12 (66.7)	5 (33.3)	p = 0.16
**Household Income, n (%)**			
< $50,000 / yr	3 (16.7)	4 (26.7)	χ2 = 0.76
$50,000–$99,999 / yr	7 (38.9)	4 (26.7)	df = 2
≥ $100,000 / yr	8 (44.4)	7 (46.7)	p = 0.68

For all infants (n = 33), the mean gestational age at birth was 39.5±1.2 weeks. The infant sample was evenly distributed by sex with 55% male and 45% female (Table 2). Infants were 39.8 ± 7.5 days old (about 5.5 weeks) when measured. Infant % fat mass was significantly different between infants born to women with or without overweight and obesity (p = 0.05). However this was due to the difference in age at the study visit (p = 0.05). After adjusting for infant age, infant growth measures did not differ significantly between maternal BMI groups.

**Table 2:** Characteristics of infant participants (N = 33).

	Infants born to mothers with BMI < 25	Infants born to mothers with BMI ≥ 25	Statistic
**No**.	18	15	
**Sex, n (%)**			
Male	9 (50.0)	9 (60.0)	χ2 = 0.33
			df = 1
Female	9 (50.0)	6 (40.0)	p = 0.57
**Race n(%)**			
Caucasian	14 (77.8)	11 (73.3)	χ2 = 8.2
African American	0 (0.0)	0 (0.0)	df = 1
Other/More than one race	4 (22.2)	4 (26.7)	p = 0.60
**Age, days**	42.1 ± 7.5	36.9 ± 6.7	t = 2.1, p <0.05
Weight, grams*	4,751.7 ± 683.0	4,303.5 ± 682.9	F(1,30) = 0.56, p = 0.46
Length, cm*	55.0 ± 2.0	55.1 ± 2.4	F(1,30) = 1.2, p = 0.28
Head Circumference, cm*	38.0 ± 1.5	37.1 ± 1.3	F(1,30) = 0.28, p = 0.60
% Fat Mass*	22.0 ± 4.9	18.0 ± 5.6	F(1,30) = 1.0, p = 0.32

* Comparisons made with age of infant as covariate.

### Inflammatory markers and hormones

#### Serum and breastmilk

For all subjects, leptin and TNF-α were significantly lower in breastmilk compared to serum (1.9±0.3 pg/mL vs. 2.3±0.4 pg/mL, p<0.001 and 0.8±0.1 pg/mL vs. 0.9±0.1 pg/mL, p = 0.03, respectively). Group 2 women had statistically higher serum and breastmilk leptin than Group 1, as well as higher levels of IL-6 in serum but not in breastmilk (Table 3). No statistically significant differences were observed in insulin, TNF-α, and IL-6 by pre-pregnancy BMI group. Serum leptin was positively correlated to breastmilk leptin concentrations for all women (β = 1.0, R^2^ = 0.80, p<0.001).

**Table 3:** Mean (log-transformed) leptin (pg/mL), insulin (μU/mL), TNF-α (pg/mL) and IL-6 (pg/mL) in blood and breastmilk at 4–8 weeks postpartum compared by pre-pregnancy weight groups.

	BMI < 25	BMI ≥ 25	Statistic
**No**.	18	15	
**Serum**			
Leptin	2.17 ± 0.34	2.57 ± 0.24	t = -3.8, p<0.01
Insulin	1.19 ± 0.49	1.22 ± 0.46	t = -0.2, p = 0.85
TNF-α	0.86 ± 0.09	0.88 ± 0.08	t = -0.6, p = 0.59
IL-6	0.63 ± 0.04	0.74 ± 0.11	t = -3.9, p<0.001
**Whole Breastmilk**			
Leptin	1.72 ± 0.38	2.10 ± 0.30	t = -3.0, p<0.01
Insulin	1.25 ± 0.37	1.52 ± 0.46	t = -1.8, p = 0.07
TNF-α	0.84 ± 0.08	1.52 ± 0.46	t = 1.0, p = 0.42
IL-6	0.82 ± 0.36	0.74 ± 0.22	t = 0.8, p = 0.40

### Polyunsaturated fatty acids

#### Plasma

Total n-6 and n-3 PUFAs comprised 43.7±4.6% and 2.4±0.8% of all fatty acids in plasma, respectively. The individual PUFAs linoleic acid (18:2) and docosahexaenoic acid (22:6) were the most abundant n-6 and n-3 PUFAs (34.5±4.0% and 1.2±0.7%, respectively). Total n-3, total n-6 and n-6/n-3 PUFA ratio did not differ between pre-pregnancy BMI groups (Table 4). In regard to individual PUFAs, Group 2 had significantly higher EDA (20:2n-6; p<0.05) and DGLA (20:2n-6; p<0.01) than Group 1.

**Table 4:** Individual n-6, n-3 polyunsaturated fatty acids (PUFAs), total n-6 PUFAs, total n-3 PUFAs and n-6/n-3 PUFA ratio expressed as a percent of contents at 4–8 weeks postpartum as compared by pre-pregnancy BMI group.

	Plasma			Whole breastmilk		
	BMI < 25	BMI ≥ 25	Statistic	BMI < 25	BMI ≥ 25	Statistic
**No**.	18	15		18	15	
**n-6 PUFAs**						
18:2 (LA)	34.0 ± 4.63	35.1 ± 3.00	t = -0.8, p = 0.43	16.8 ± 4.57	17.9 ± 2.91	t = -0.7, p = 0.46
18:3 (GLA)	0.36 ± 0.21	0.27 ± 0.10	t = 1.7, p = 0.10	0.15 ± 0.05	0.11 ± 0.04	t = 2.4, p<0.05
20:2 (EDA)	0.26 ± 0.07	0.31 ± 0.06	t = -2.1. p<0.05	0.33 ± 0.08	0.39 ± 0.09	t = -2.1, p<0.05
20:3 (DGLA)	1.17 ± 0.58	1.73 ± 0.51	t = -2.9, p<0.01	0.38 ± 0.08	0.43 ± 0.11	t = -1.6, p = 0.26
20:4 (AA)	6.84 ± 1.56	6.52 ± 1.65	t = 0.6, p = 0.57	0.47 ± 0.08	0.48 ± 0.12	t = -0.5, p = 0.66
20:5	0.09 ± 0.07	0.06 ± 0.05	t = 1.1, p = 0.28	0.05 ± 0.08	0.05 ± 0.03	t = -0.7, p = 0.49
22:2	0.25 ± 0.26	0.12 ± 0.15	t = 1.8, p = 0.08	0.03 ± 0.08	0.04 ± 0.03	t = -0.04, p = 0.97
22:4	0.22 ± 0.09	0.28 ± 0.08	t = -2.0, p = 0.06	0.08 ± 0.08	0.09 ± 0.02	t = -1.4, p = 0.17
22:5	0.01 ± 0.01	0.02 ± 0.01	t = -1.3, p = 0.21	0.00 ± 0.08	0.01 ± 0.00	t = -1.6, p = 0.11
**Total n-6**	43.2 ± 5.36	44.4 ± 3.60	t = -0.7, p = 0.47	18.3 ± 4.72	19.5 ± 3.08	t = -0.8, p = 0.43
**n-3 PUFAs**						
18:3 (ALA)	0.48 ± 0.11	0.56 ± 0.18	t = -1.7, p = 0.10	1.09 ± 0.54	1.04 ± 0.36	t = 0.3, p = 0.76
18:4 (SDA)	Undetectable	Undetectable	-	0.03 ± 0.02	0.02 ± 0.02	t = 1.1, p = 0.30
20:4 (ETA)	0.02 ± 0.07	0.05 ± 0.13	t = -0.9, p = 0.35	0.01 ± 0.03	0.07 ± 0.06	t = -3.5, p<0.01
20:5 (EPA)	0.24 ± 0.15	0.17 ± 0.07	t = 1.6, p = 0.11	0.01 ± 0.02	Undetectable	-
22:5 (DPA)	0.49 ± 0.23	0.38 ± 0.14	t = 1.5, p = 0.14	0.14 ± 0.05	0.13 ± 0.05	t = 0.5, p = 0.62
22:6 (DHA)	1.10 ± 0.64	1.25 ± 0.68	t = -0.7, p = 0.49	0.13 ± 0.06	0.18 ± 0.12	t = -1.5, p = 0.14
**Total n-3**	2.31 ± 0.86	2.42 ± 0.81	t = -0.4, p = 0.72	1.40 ± 0.58	1.43 ± 0.50	t = -0.1, p = 0.91
**n-6/n-3 ratio**	21.0 ± 7.41	20.8 ± 8.42	t = 0.1, p = 0.95	14.0 ± 3.35	14.9 ± 4.57	t = -0.7, p = 0.51

#### Breastmilk

The most abundant individual n-6 and n-3 PUFAs in breastmilk were linoleic acid (LA) (17.3±3.9%) and alpha-linolenic acid (ALA) (1.1±0.5%), respectively. Total n-3 PUFAs accounted for 1.4±0.5% of total fatty acids in breastmilk as compared to n-6 PUFAs, which accounted for 18.8±4.0% of total fatty acids. Both total n-3 PUFAs and n-6 PUFAs were significantly lower in breastmilk than in plasma at 4–8 weeks postpartum (1.4±0.5% vs. 2.6±0.8%, p<0.001 and 4.6±0.8% vs. 4.0±0.7%, p<0.001, respectively). Similarly, n-6/n-3 PUFA ratio was significantly lower in breastmilk than in plasma (3.9±0.7 vs. 7.8±1.4, p<0.001). No significant differences were observed in total n-3, total n-6 and n-6/n-3 PUFA ratio between pre-pregnancy BMI groups. Group 1 had higher levels of n-6 PUFA gamma-linolenic acid (GLA) as compared to Group 2 (0.15±0.05 vs. 0.11±0.04, p<0.05) whereas Group 2 had higher levels of n-6 PUFA eicosadienoic acid (EDA) (0.39±0.09 vs. 0.33±0.08, p<0.05) and n-3 PUFA eicosatetraenoic acid (ETA) as compared to Group 1 (0.07±0.06 vs. 0.01±0.03, p<0.01) (Table 4).

### Relationship between inflammatory markers and hormones and PUFAs

#### Serum inflammatory markers and hormones and plasma PUFAs

**Fig 1:**
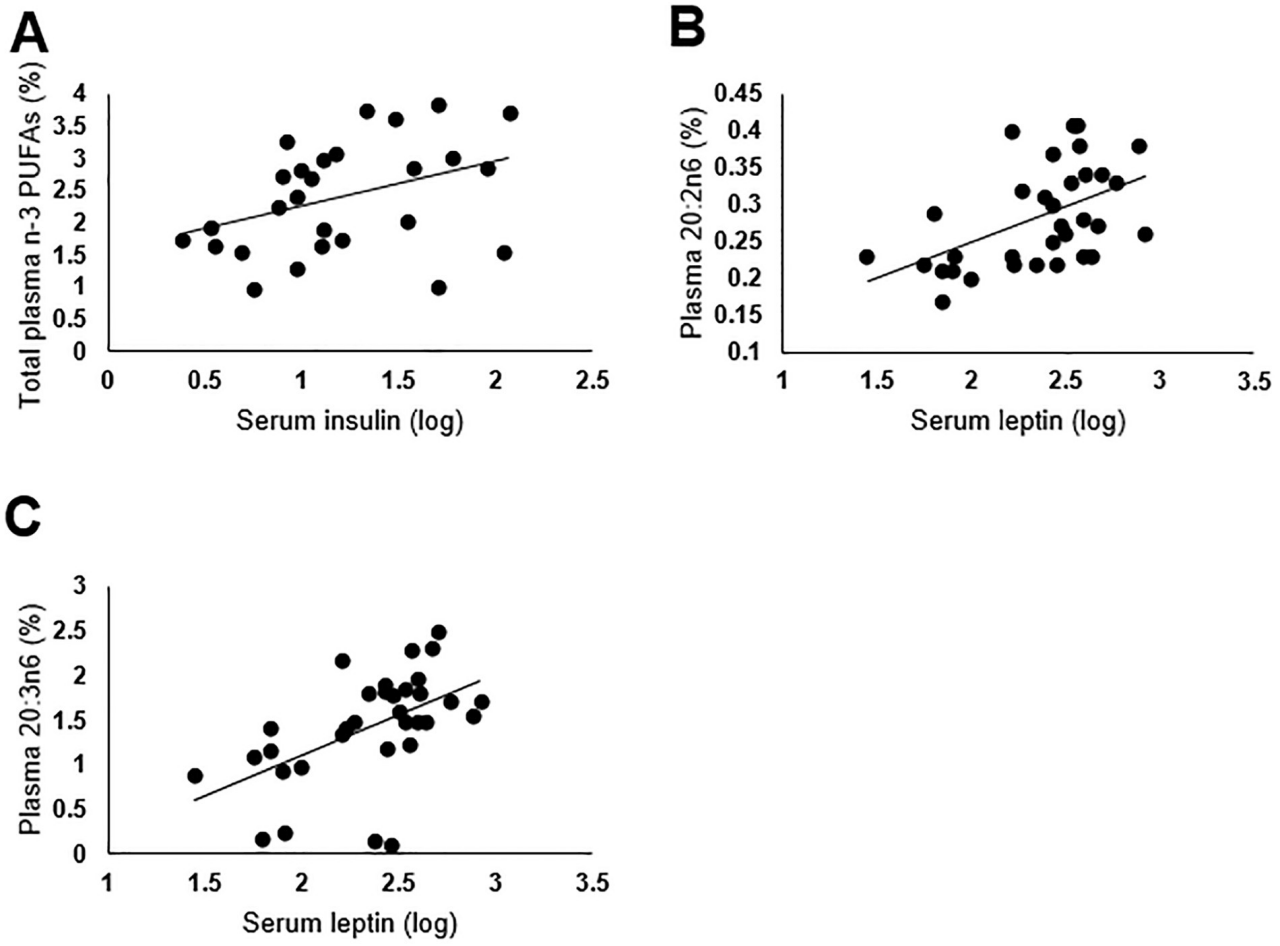
Correlations between serum hormones and plasma polyunsaturated fatty acids (PUFAs) at four to eight weeks postpartum. (A) Serum insulin and total plasma n-3 PUFAs. (B) Serum leptin and plasma 20:2n6. (C) Serum leptin and plasma 20:3n6.

**Fig 2:**
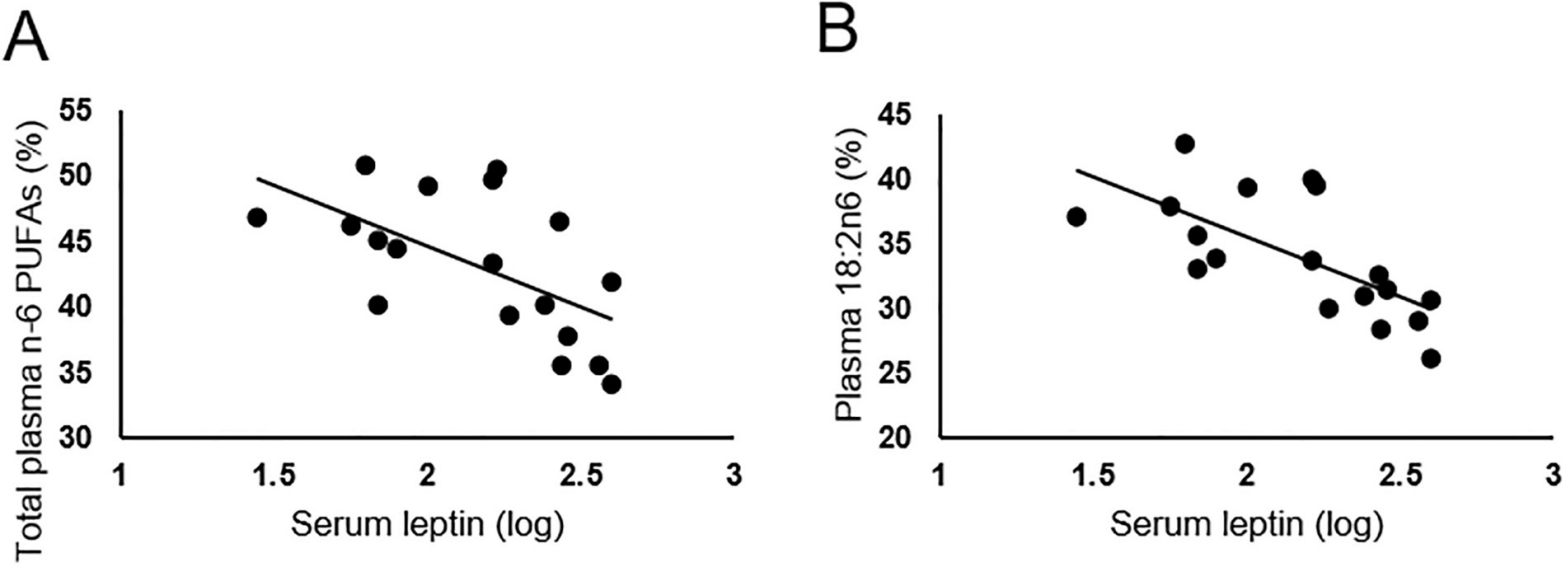
Correlations between serum leptin and plasma n-6 polyunsaturated fatty acids (PUFAs) at four to eight weeks postpartum among women without overweight or obesity. (A) Serum leptin and total plasma n-6 PUFAs. (B) Serum leptin and plasma 18:2n6.

Based on the hypothesis that higher circulating levels of n-6 PUFAs would be correlated with higher pro-inflammatory markers, we measured correlations between plasma PUFAs and serum biomarkers. For the entire sample, total n-3 PUFAs were positively correlated with insulin (β = 0.21, R^2^ = 0.15, p = 0.05) (Fig 1a). No inflammatory marker or hormone was statistically correlated with circulating n-6/n-3 PUFA ratio. Considering individual PUFAs, both 20:2n6 (EDA) and 20:3n6 (DGLA) were positively correlated to leptin (β = 2.71, R^2^ = 0.26, p = .01 and β = 0.31, R^2^ = 0.29, p<0.01, respectively) (Fig 1b and 1c). When comparing by pre-pregnancy BMI group, total n-6 PUFAs were negatively correlated to leptin in Group 1 (β = -0.04, R^2^ = 0.28, p = 0.05) (Fig 2a). Also, 18:2 n-6 (LA) was negatively correlated to leptin (β = -0.05, R^2^ = 0.46, p = 0.01) in Group 1 (Fig 2b).

#### Breastmilk inflammatory markers and hormones and plasma PUFAs

Correlations did not differ between total n-3 PUFAs and total n-6 PUFA between pre-pregnancy BMI groups. The n-6/n-3 PUFA ratio was not correlated with inflammatory marker and hormone concentrations measured in breastmilk. Among Group 1 women, n-6 PUFA 18:3 n-6 (GLA) was positively correlated to IL-6 (β = 1.11, R^2^ = 0.40, p = 0.02). Among Group 2 women, the individual PUFA 20:4 n-6 (AA) was positively correlated to TNF-α (β = 0.56, R^2^ = 0.45, p = 0.02).

#### Breastmilk inflammatory markers and hormones and whole breastmilk PUFAs

No significant correlations were observed between total n-3 PUFAs, total n-6 PUFAs and n-6/n-3 PUFA ratio and inflammatory markers and hormones in breastmilk in all women or by pre-pregnancy BMI group. After applying false discover rate methodology, the individual PUFA 20:3n6 (DGLA) was positively correlated with leptin (β = 1.81, R^2^ = 0.24, p = 0.02) for the entire sample. No individual PUFAs were significantly correlated with insulin, TNF-α and IL-6. When compared by BMI groups, gamma-linoleic acid (18:3n6) was negatively correlated with insulin (β = -3.59, R^2^ = 0.50, p<0.05) and positively correlated with TNF-α (β = 0.60, R^2^ = 0.39, p<0.05) among women with overweight and obesity only. Also, 22:6n3 (DHA) was positively correlated with TNF-α (β = 0.31, R^2^ = 0.39, p = 0.05) in women with overweight and obesity.

#### Breastmilk inflammatory markers and hormones and infant growth measures

After controlling for infant age at the 4–8 weeks visit, breastmilk biomarkers were all negatively correlated with infant growth measures except for TNFα, which was positively correlated with infant weight and % fat mass (Table 5). When maternal pre-pregnancy BMI groups were assessed separately, these negative correlations were only observed among Group 1 infants, the exception being that TNFα was also negatively correlated with infant weight and % fat mass (β = -2860, R^2^ = 0.54, p<0.01 and β = -9.78, R^2^ = 0.96, p<0.001, respectively). No significant correlations between breastmilk biomarkers and infant growth measures were observed among infants born to mothers with overweight or obesity.

**Table 5:** Linear regressions with breastmilk inflammatory markers and hormones and infant growth measures for all infants.

	**Infant length**		
	B	R^2^	p-value
**Leptin**	-1.34	0.14	0.12
**Insulin**	-0.03	0.06	0.42
**TNF-α**	1.36	0.13	0.12
**IL-6**	-2.1	0.21	0.03
	**Infant weight**		
	B	R^2^	p-value
**Leptin**	-650.1	0.49	<0.001
**Insulin**	-184.1	0.37	<0.01
**TNF-α**	101.5	0.40	<0.001
**IL-6**	-541.5	0.45	<0.001
	**Infant head circumference**		
	B	R^2^	p-value
**Leptin**	-1.10	0.42	<0.001
**Insulin**	-0.55	0.34	<0.01
**TNF-α**	-1.36	0.39	<0.01
**IL-6**	-1.45	0.46	<0.001
	**Infant % fat mass**		
	B	R^2^	p-value
**Leptin**	-3.12	0.45	<0.001
**Insulin**	-0.42	0.43	<0.001
**TNF-α**	2.36	0.45	<0.001
**IL-6**	-2.94	0.47	<0.001

#### Breastmilk PUFAs and infant growth measures

For all infants, total n-6 PUFAs (β = -34.2, R^2^ = 0.44, p<0.001), total n-3 PUFAs (β = -62.7, R^2^ = 0.41, p<0.001) and the n-6/n-3 PUFA ratio (β = -35.2, R^2^ = 0.44, p<0.001) were negatively correlated with infant weight. Total n-6 PUFAs were negatively correlated to head circumference (β = -0.06, R^2^ = 0.40, p<0.001) and % fat mass (β = -0.17, R^2^ = 0.46, p<0.001). The n-6/n-3 PUFA ratio was also negatively correlated to head circumference (β = -0.11, R^2^ = 0.46, p<0.001) and % fat mass (β = -0.35, R^2^ = 0.50, p<0.001). In contrast, total n-3 PUFAs was positively correlated with both head circumference and % fat mass (β = 0.03, R^2^ = 0.38, p = 0.001, and β = 0.03, R^2^ = 0.38, p = 0.001, respectively). Length was the only infant growth parameter with no association with breastmilk PUFAs. When compared by maternal pre-pregnancy BMI group, no correlations were observed between breastmilk PUFAs and Group 2 infant growth measures. For Group 1 infants, each breastmilk PUFA outcome (total n-6 PUFAs, total n-3 PUFAs, and n-6/n-3 PUFA ratio) was negatively correlated with weight, length, head circumference, and % fat mass (Table 6).

**Table 6:** Linear regressions with breastmilk polyunsaturated fatty acids (PUFAs) and infant growth measures for infants born to women without overweight or obesity (n = 18).

	**Infant length**		
	B	R^2^	p-value
**n-6/n-3 PUFA ratio**	-0.05	0.40	0.02
**Total n-6**	-0.08	0.43	0.01
**Total n-3**	-0.40	0.40	0.02
	**Infant weight**		
	B	R^2^	p-value
**n-6/n-3 PUFA ratio**	-31.7	0.45	0.01
**Total n-6**	-50.9	0.55	<0.01
**Total n-3**	-198.0	0.49	0.01
	**Infant head circumference**		
	B	R^2^	p-value
**n-6/n-3 PUFA ratio**	-0.08	0.54	<0.01
**Total n-6**	-0.07	0.56	<0.01
**Total n-3**	-0.26	0.52	<0.01
	**Infant % fat mass**		
	B	R^2^	p-value
**n-6/n-3 PUFA ratio**	-0.23	0.68	<0.001
**Total n-6**	-0.18	0.69	<0.001
**Total n-3**	-0.07	0.66	<0.001

## Discussion

In this novel, prospective cohort study, we assessed the interrelationships between three categories of bioactive compounds, including hormones, cytokines and polyunsaturated fatty acids (PUFAs), both in circulation and in breastmilk one cohort of 33 women in early postpartum. We further compared the relative concentrations of these compounds in women with a pre-pregnancy BMI < 25 (Group 1) vs. a pre-pregnancy of BMI ≥25 (Group 2). Secondarily, this study also sought to characterize the relationship between these bioactive substances and measures of infant growth. To our knowledge, this is the first study to simultaneously study these specific compounds in the same cohort of mother/infant dyads at the same time.

We hypothesized that Group 2 women would have a more obesogenic profile with regards to the bioactive hormones and cytokines we measured, both in circulation and in breastmilk. We observed that serum leptin, but not insulin, was higher in Group 2 women. Other studies have also reported that blood concentrations of leptin is associated with mothers with a BMI ≥ 25 up to two weeks postpartum [26–29]. Group 2 women also had more systemic inflammation, with higher levels of circulating interleukin-6 (IL-6) versus Group 1. This finding is similar to a study by Christin and Porter who reported that obesity predicted higher serum IL-6 at four to six weeks postpartum in a sample of 57 women in the U.S. [Christian 2014 26] Likewise, a study of non-postpartum, healthy African American women noted that higher serum IL-6 was positively associated with BMI [30]. However, serum TNF-α in the current study did not vary by pre-pregnancy BMI group, a finding which has been observed in other similar studies [29–32].

We measured the concentrations of nine individual n-6 and six n-3 PUFA species to comprehensively calculate n-6/n-3 PUFA ratios with the assumption that some samples would have a higher n-6/n-3 ratio, which is considered a pro-inflammatory profile [33]. Interestingly, the total n-6 and n-3 PUFAs n-6/n-3 ratios were not statistically different between weight groups. Comparing our results to other studies has limitations since no other published study has included as many individual PUFAs and have only focused on breastmilk concentrations (and not in plasma) in the postpartum period [33–35]. For example, Panagos and colleagues measured total n-6 PUFAs as the sum of 18:2 (LA) and 20:4 (AA) and total n-3 PUFAs as the sum of 18:2 (ALA), 20:5 (EPA), 22:5 (DPA) and 22:6 (DHA) in breastmilk at two months postpartum in a sample of 21 U.S. women without and 21 with overweight or obesity [33]. Rudolph and colleagues measured AA/DHA + EPA ratios in breastmilk in 26 women without overweight or obesity, 12 with overweight and 10 with obesity at two weeks and four months postpartum [34], while another study assessed LA + DGLA + AA/ALA + EPA + DHA ratios in breastmilk in 41 women without overweight obesity vs. 41 women with overweight or obesity at three and 10 days and one and two months postpartum [35]. In each of these studies, n-6/n-3 breastmilk PUFA ratios were statistically higher in women with overweight or obesity [33, 35], and BMI was moderately correlated with n-6/n-3 breastmilk PUFA ratios [34]. As a comparison, in the current investigation n-6/n-3 ratios were calculated using the same individual PUFA combinations as the aforementioned studies. Results were similar indicating that PUFA ratios were higher in women with overweight or obesity using the same PUFAs described by Storck Lindholm and colleagues [35]; however, the current findings did not reach statistical significance. Cumulatively, these findings suggest that further research to determine the association between individual and total PUFA profiles and weight status is warranted.

Total circulating n-6 and n-3 PUFAs did not correlate with total n-6 and n-3 PUFAs in breastmilk, regardless of pre-pregnancy BMI. While we did not observe differences with regards to n-6 and n-3 totals or ratios, there were notable differences between individual PUFAs and weight groups. For example, Group 2 women (BMI≥25) versus Group 1 women (BMI<25) had lower breastmilk concentrations of 18:3n-6 (GLA) and 20:5n-3 (EPA) and higher levels of 20:2n-6 (EDA) and 20:4n-3 (ETA). In comparison, in a Swedish study, 41 women with obesity (BMI ≥ 30 kg/m2) had lower breastmilk concentrations of EPA and DHA at three days postpartum compared to 41 women without obesity. However, these differences were not maintained two months later [35]. Similarly, a U.S. study reported lower breastmilk concentrations of n-3 PUFAs ALA, EPA, DPA and DHA at two months postpartum in 21 women with overweight or obesity compared to those without overweight or obesity. While human breastmilk is considered mature at six weeks postpartum [36], these findings suggest that the breastmilk of women with overweight or obesity may be compromised by a pro-inflammatory lipid profile. However, additional studies are needed to confirm this observation.

Mechanistically, n-6 and n-3 PUFAs serve as metabolic precursors to lipid mediators that can either upregulate or downregulate the production of pro-inflammatory cytokines through macrophages in adipocytes [16, 17, 37–39]. Based on this, we assessed whether n-6 and n-3 PUFAs and were correlated with TNF-α and IL-6 in circulation and in breastmilk. No significant relationships were noted between total n-6, n-3 or total n-6/n-3 PUFA ratios and pro-inflammatory cytokines either in circulation or in breastmilk regardless of weight status. In contrast, a Canadian study of 47 normal weight (BMI<25) women reported that a higher breastmilk n-6/n-3 PUFA ratio was positively correlated to TNF-α and IL-6 breastmilk concentrations [40]. However, the Canadian breastmilk samples were collected within two weeks postpartum as compared to four to eight weeks in our study. These findings suggest that any influence PUFAs would otherwise have on TNF-α and IL-6 may have been mitigated beyond two weeks postpartum.

There were some interesting findings for individual PUFAs. For example, plasma concentrations of the n-6 PUFA GLA were positively associated with breastmilk IL-6 among women without overweight or obesity. Alternatively, plasma AA was positively associated with breastmilk TNF-α in women with overweight or obesity. Thus, maternal pre-pregnancy BMI specifically moderates the influence of certain individual PUFAs either in circulation or breastmilk. This finding is similar to a study in over 2,800 African American, Chinese, Hispanic and White non-pregnant adults, which concluded that obesity modified the association between plasma PUFAs and IL-6 [41]. Specifically, the researchers reported that plasma DGLA was positively associated with IL-6 only in participants with obesity. Collectively, this work highlights the need for future studies to include lipidomic analysis for a more complete examination of lipid species in breastmilk.

Despite the relative homogeneity of breastmilk contents between Group 1 and Group 2 women, the most striking results we observed were in relation to breastmilk and infant growth measures when compared by prepregnancy weight groups. The only statistically significant associations between measured breastmilk components and infant growth measures were observed among Group 1 infants, or infants born to mothers with a pre-pregnancy BMI < 25. A possible explanation for these observations is consistent with fetal programming whereby infants who were gestated by mothers with a BMI ≥ 25 are predisposed to be less responsive to breastmilk in terms of growth and development when compared to infants born to normal weight women. However, reports from other studies suggests that the relationship between mothers’ weight status, milk contents and infant growth is more complex. For example, Young et. al., reported breastmilk insulin was negatively associated with infant weight for length Z-scores from two weeks to four months postpartum only among infants gestated by women with a prepregnancy BMI≥25 [14]. The same study reported no association with infant growth and other breastmilk components, including leptin, IL-6 and TNF-α. With regards to breastmilk PUFAs, Panagos and colleagues reported that mothers with a BMI ≥ 25 had higher n-6/n-3 PUFA ratios but infant growth measures (skinfold thickness, BMI-z) did not differ at two months postpartum [33]. In addition, Rudolph and colleagues reported that infant fat mass was positively correlated with breastmilk n-6/n-3 ratio, regardless of maternal pre-pregnancy BMI [34]. It is possible that any growth differences between these infants in response to breastmilk contents may not become apparent until after two months of age.

Interestingly, leptin was the only biomarker that showed a correlation between blood and breastmilk levels of the hormones and pro-inflammatory cytokines that we studied. Furthermore, breastmilk leptin levels in Group 2 women were higher than Group 1. It is possible that leptin, well known for its role in appetite regulation, may have suppressed infants’ hunger, reduced consumption and mitigated any correlations between infant growth measures and breastmilk contents. Other studies have also reported that breastmilk leptin is positively correlated with maternal pre-pregnancy BMI and negatively correlated with infant growth measures at four and six months post-delivery [6, 42]. We are unaware of any information that describes a possible mechanism for this finding in human subjects, linking it to adverse health outcomes later in life. However, several rodent studies have shown that suckling pups of mothers who were fed high-fat diets to induce obesity were programmed to develop insulin [43–47] and leptin [46, 47] resistance and subsequent metabolic disorder, predisposing the offspring to develop obesity later in life. This effect may, in part, due to the disruption of normal hypothalamic insulin and leptin signal transduction pathways [46]. The resultant insulin and leptin resistance would likely result in unfavorable energy intake and expenditure and favor the development of obesity.

Strengths of this study include the robust phenotyping of the study participant/infant dyads and the extensive lipidomic analyses that were performed. Furthermore, we are aware of no other reports that have simultaneously documented the interrelationships between hormones, inflammatory cytokines, and PUFAs in blood and breastmilk and resultant impacts on breastfeeding infant growth measures as compared by maternal pre-pregnancy weight groups. Notable limitations of this study include the study sample size and lack of racial diversity, which may affect the generalizability of our findings to larger populations. The sample size likely impacted the statistical power of our analyses, as was evident with the wide variance in certain measures that required log transformations to non-normally distributed variables. However, such variance of relatively unstudied biomarkers in breastmilk has also been reported in larger studies [12]. Findings are also limited because we did not quantify how much formula or other substances (e.g., cow’s milk, water, juice) were used. However, in the eight cases that mothers reported that they had ever fed their infants something other than breastmilk, the majority reported that they did not regularly supplement. Although sporadic, this deviation from breast feeding may have impacted the significance of our results. Due to the scope of the study, we did not perform a macronutrient analysis of the breastmilk, which may have shown differing concentrations between weight groups. Moreover, it is not possible to determine specific macronutrient influences on infant growth measures. Lastly, it was difficult to standardize the times the milk samples and infant measures were collected due to scheduling difficulties with participants’ busy schedules. However, we did our best to control for this variance by scheduling site visits as consistently as possible and controlling for as much variance as statistically possible. Regardless, our findings add to a growing body of evidence linking maternal BMI to nutritional programming in infants who are fed breastmilk.

## Conclusions

To our knowledge, this is the first study to simultaneously demonstrate all the associations between plasma PUFAs and whey breastmilk leptin, insulin, TNF-α and IL-6, and to compare PUFAs and inflammatory markers and hormones to infant weight, length, head circumference and % fat mass at 4–8 weeks postpartum in the same cohort. The pro-inflammatory qualities of breastmilk were associated with breastfeeding infant growth measures. However, infants born to women with overweight or obesity demonstrated less responsive growth to breastmilk contents as no relationships were observed between whey inflammatory markers, hormones, PUFAs and growth measures. Conversely, TNF-α was positively related, and IL-6, leptin, insulin, total n-6, n-3 and n-6/n-3 PUFAs in whey breastmilk were negatively correlated to several infant growth measures in offspring of women without overweight or obesity. Our findings highlight the importance of understanding the role of bioactive components of human breastmilk and their impact on the future health of infants. In particular, PUFAs, inflammatory markers and hormones known to play an important role in adiposity may, therefore, impact early infant development. Future research should explore the longitudinal health outcomes of infants to determine the influence of maternal inflammatory markers and hormones and lipid species including PUFAs on growth and development throughout childhood.
